# Branch retinal artery occlusion associated with posterior uveitis

**DOI:** 10.1186/1869-5760-3-16

**Published:** 2013-01-21

**Authors:** Rim Kahloun, Samah Mbarek, Imen Khairallah-Ksiaa, Bechir Jelliti, Salim Ben Yahia, Moncef Khairallah

**Affiliations:** 1 Department of Ophthalmology, Fattouma Bourguiba University Hospital, Monastir 5019, Tunisia; 2 Faculty of Medicine, University of Monastir, Monastir 5019, Tunisia

**Keywords:** Branch retinal artery occlusion, Posterior uveitis, Fluorescein angiography, Visual impairment

## Abstract

**Background:**

The purpose of this study is to report the clinical features and visual outcome of branch retinal artery occlusion (BRAO) associated with posterior uveitis. This is a retrospective study including the 18 eyes of 18 patients. All patients underwent a complete ophthalmic evaluation. Fundus photography, fluorescein angiography, and visual field testing were performed in all cases.

**Results:**

Diseases associated with BRAO included active ocular toxoplasmosis in 7 patients, rickettsiosis in 4, Behçet’s uveitis in 2, West Nile virus infection in 1, idiopathic retinal vasculitis in 1, Crohn’s disease in 1, ocular tuberculosis in 1, and idiopathic retinal vasculitis, aneurysms, and neuroretinitis syndrome in 1 patient. The mean initial visual acuity was 20/50. BRAO involved the first order retinal artery in 33.3% of the eyes, the second order retinal artery in 33.3%, an arteriole in 27.8%, and a cilioretinal artery in 5.5%. The macula was involved in 44.4% of the eyes and an acute focus of retinitis or retinochoroiditis was associated to BRAO in 55.5%. Repermeabilization of the occluded artery occurred in all patients with permanent scotomas in the corresponding visual field. The mean visual acuity at last visit was 20/32.

**Conclusions:**

BRAO, with subsequent visual impairment, may occur in the eyes with posterior uveitis. Physicians should be aware of such vision-threatening complication of infectious and inflammatory eye diseases.

## Background

Infectious or inflammatory eye diseases may result in numerous vascular complications. These mainly include retinal hemorrhages, retinal vascular hyperpermeability, retinal vascular occlusion, macroaneurysms, retinal or choroidal neovascularization, and retinochoroidal anastomosis [1,2]. Although branch retinal vein occlusion (BRVO) is considered to be a common complication of posterior uveitis associated with retinal vasculitis, data on the inflammatory branch retinal artery occlusion (BRAO) are relatively scarce. In this study we describe the clinical features and visual outcome of BRAO associated with posterior uveitis.

## Results

Ten patients (55.5%) were men, and eight patients (44.5%) were women. The age of our patients ranged from 18 to 56 years (mean 37.8; median 37.5).

**Figure 1:**
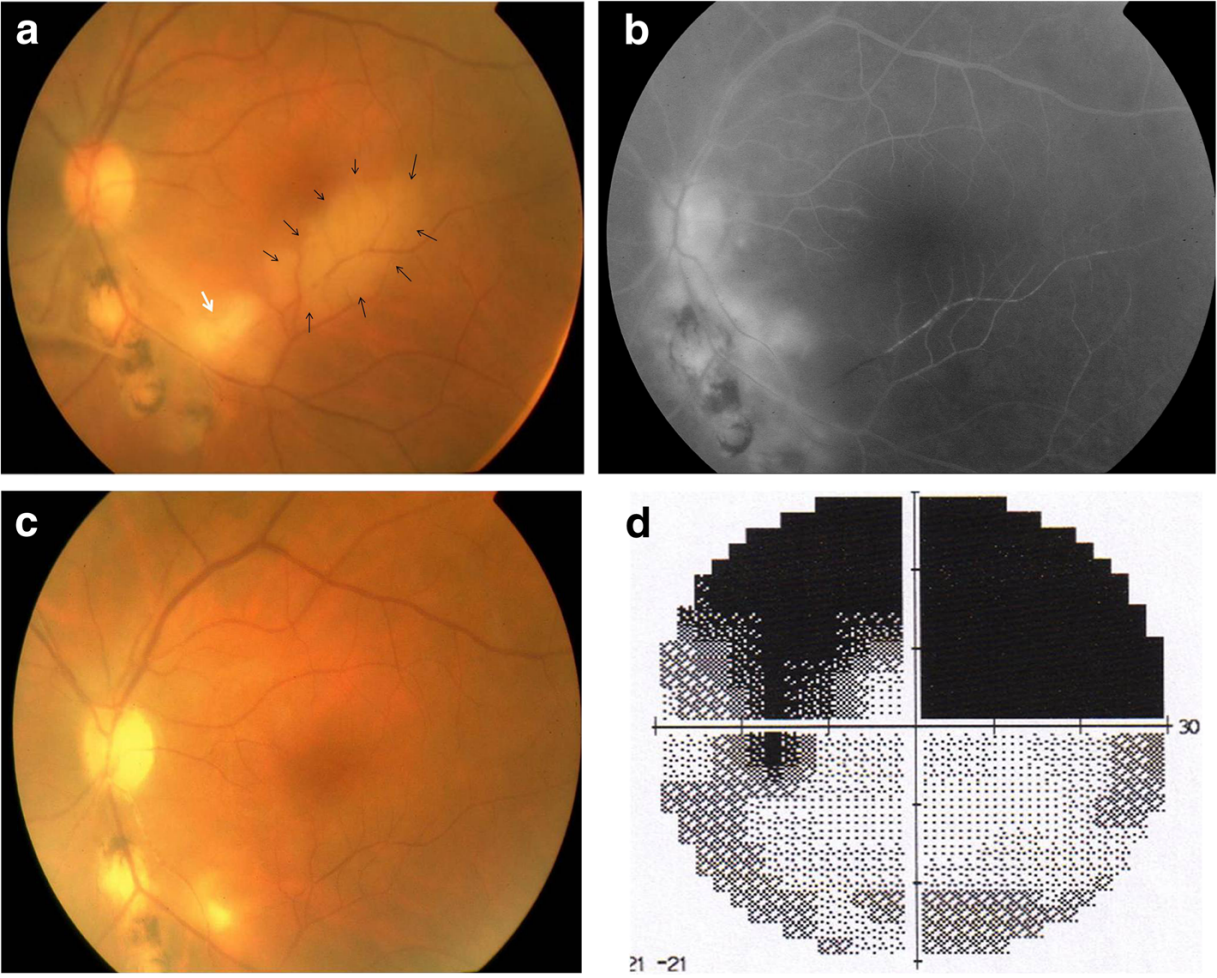
**Branch retinal artery occlusion associated with ocular toxoplasmosis.** (**a**) Color fundus photograph of the left eye of a patient with ocular toxoplasmosis shows an active focus of retinochorioretinitis (white arrow) adjacent to old pigmented scars infero-temporally and an area of retinal whitening along the inferior temporal arcade (black arrows). (**b**) Early phase fluorescein angiogram shows delayed filling of inferior temporal branch retinal artery, capillary nonperfusion corresponding to the area of retinal whitening, and hypofluorescence of the focus of retinochoroiditis. (**c**) Color fundus photograph 3 months later shows resolution of the retinal whitening. Note the presence of a persistent scotoma on automated perimetry (**d**).

**Figure 2:**
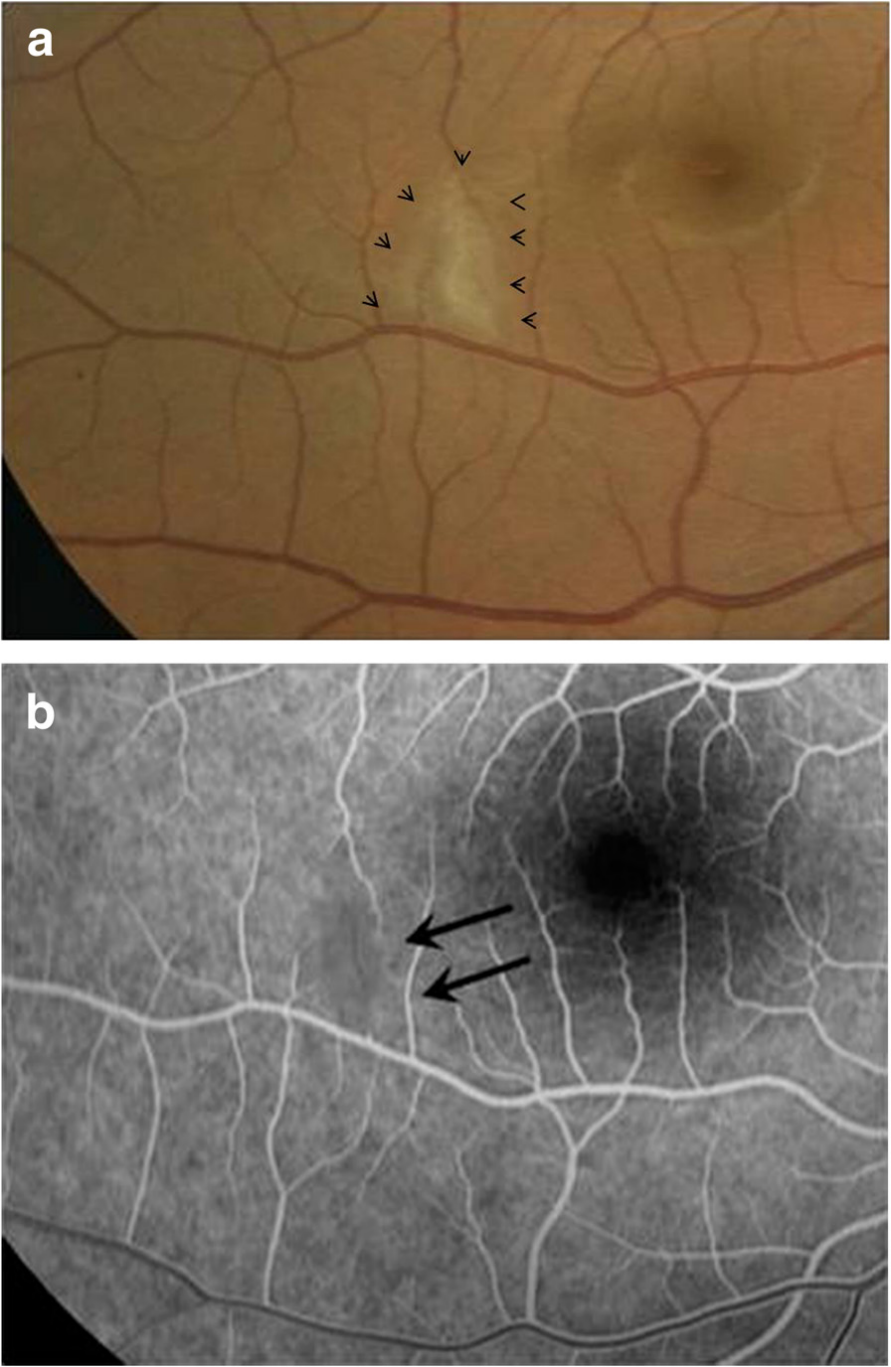
**Branch retinal artery occlusion associated with rickettsiosis.** (**a**) Color fundus photograph of the right eye of a patient with rickettsiosis shows an area of retinal whitening sparing the fovea (arrow heads). (**b**) Early phase fluorescein angiogram confirms the diagnosis of branch retinal arteriolar occlusion sparing the fovea (black arrows).

Diseases associated with BRAO were active ocular toxoplasmosis in 7 patients (7.5% of all toxoplasmosis cases recorded in our department) (Figure 1), rickettsiosis (Mediterranean spotted fever) in 4 patients (4.2% of all Mediterranean spotted fever cases recorded in our department) (Figure 2), Behçet’s uveitis in 2 patients (1.3% of all Behçet’s uveitis cases recorded in our department), West Nile virus infection in 1 patient (2.4% of all cases of West Nile virus infection with posterior uveitis recorded in our department), idiopathic retinal vasculitis in 1 patient, Crohn’s disease in 1 patient, ocular tuberculosis in 1 patient (4% of all ocular tuberculosis cases recorded in our department) and idiopathic retinal vasculitis, aneurysms, and neuroretinitis (IRVAN) syndrome in 1 patient (Table 1).

**Table 1:** Demographic and clinical characteristics of our patients

**Characteristics**	**Values**
Number of patients (eyes)	18 (18)
Age (years)	
Range	18-56
Mean	37.8
Median	37.5
Gender (*n*) (%)	
Male	10 (55.5)
Female	8 (44.5)
Associated inflammatory eye disease (*n*) (%)	
Ocular toxoplasmosis	7 (38.9)
Rickettsiosis	4 (22.2)
Behçet’s disease	2 (11.1)
Ocular tuberculosis	1 (5.5)
IRVAN syndrome	1 (5.5)
Crohn disease	1 (5.5)
Idiopathic vasculitis	1 (5.5)
West Nile virus infection	1 (5.5)
Cat scratch disease	0 (0)
Site of occlusion (*n*) (%)	
First order retinal artery	6 (33.3)
Second order retinal artery	6 (33.3)
Arteriole	5 (27.8)
Cilioretinal artery	1 (5.5)
Initial visual acuity	
Range	20/400-20/20
Mean	20/50
Median	20/40
Visual acuity at last visit	
Range	20/200-20/20
Mean	20/32
Median	20/25

IRVAN, idiopathic retinal vasculitis, aneurysms, and neuroretinitis syndrome.

Initial visual acuity (VA) ranged from 20/400 to 20/20 (mean, 20/50; median, 20/40). Fundus examination showed the focal area of retinal whitening corresponding to the occluded artery in all cases. An acute focus of retinitis or retinochoroiditis was associated to BRAO in 10 eyes (55.5%), including 7 eyes with ocular toxoplasmosis, 2 eyes with rickettsiosis, and 1 eye with Behçet’s uveitis. The occluded vessel passed through the area of acute retinitis or retinochoroiditis in all these cases.

Fluorescein angiography (FA) revealed delayed filling of the occluded branch retinal artery and capillary nonperfusion corresponding to the area of retinal whitening seen clinically in all cases.

BRAO involved a first order retinal artery in 6 eyes (33.3%), a second order retinal artery in 6 eyes (33.3%), an arteriole in 5 eyes (27.8%) and a cilioretinal artery in 1 eye (5.5%). The macula was involved in 8 eyes (44.4%).

**Figure 3:**
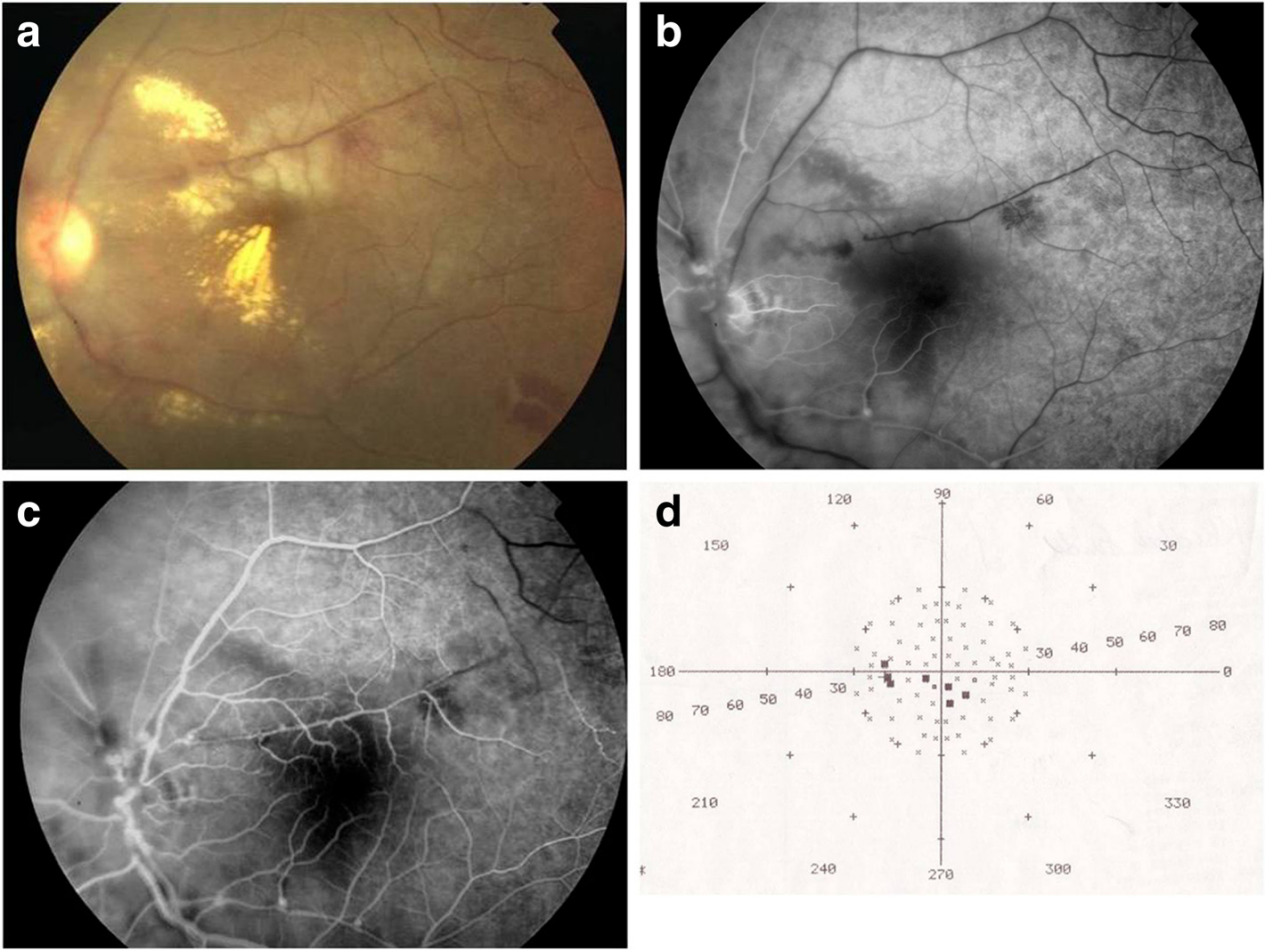
**Branch retinal artery occlusion associated with IRVAN syndrome.** (**a**) Color fundus photograph of the left eye of a patient with IRVAN syndrome shows an area of retinal whitening along upper temporal vessel. Early phase (**b**) and mid-phase (**c**) fluorescein angiograms show branch retinal artery occlusion. Note the presence of multiple macroaneurysms on the course of this artery. (**d**) Visual field testing shows multiple central scotomas in the area corresponding to the occluded artery.

A cilioretinal artery occlusion associated with BRVO was recorded in one patient with Behçet’s uveitis. A BRAO associated with BRVO was recorded in one eye with tuberculous uveitis. In the eye with IRVAN syndrome, BRAO occurred at the level of an aneurysm and was not associated to any prior laser photocoagulation (Figure 3). Optical coherence tomography (OCT) showed serous retinal detachment in 4 of the 8 eyes (50%).

Patients with ocular toxoplasmosis were treated with a combination of pyrimethamine (100 mg the first day then 50 mg daily), azithromycin (500 mg the first day then 250 mg daily), and prednisone for 4 to 6 weeks. Patients with rickettsiosis were treated with a 2-week course of oral doxycycline. Patients with Behçet’s uveitis were treated with systemic corticosteroids associated with immunosuppressive therapy (azathioprine for the first patient and a combination of azathioprine and cyclosporine for the second patient). Patient with ocular tuberculosis was treated with antituberculous therapy (four drugs for 2 months followed by two drugs for 7 months) associated with oral corticosteroids. Patients with idiopathic retinal vasculitis, Crohn’s disease, and IRVAN syndrome were treated with oral corticosteroids.

During follow-up, all foci of retinitis or retinochoroiditis became inactive. Repermeabilization of the occluded artery occurred in all patients with residual scotoma in the corresponding visual field (Figure 1). Retinal neovascularisation was recorded in one eye (patient with West Nile virus infection). VA at last visit ranged from 20/200 to 20/20 (mean, 20/32; median, 20/25).

## Discussion

Most publications on inflammatory BRAO consisted of single-case reports, and to our knowledge, our study is the largest case series reported to date. Our findings, consistent with those from previous reports, show that ocular toxoplasmosis is the leading cause of inflammatory BRAO [3-10], followed by an array of other inflammatory infectious or noninfectious retinochoroidal disorders [11-22]. Rickettsial infection was found to be the second most common cause of BRAO in our series. Mediterranean spotted fever, a rickettsial disease caused by *Rickettsia conorii*, is endemic in the Mediterranean region including North Africa [11]. Retinal vascular involvement in this disease is common due to a marked tropism of the rickettsial organisms in the small blood vessels throughout the body, including the retina [11]. Cat scratch disease, an infectious disease caused by the gram-negative bacteria *Bartonella henselae* that has a similar tropism in the retinal vessels has been previously associated with BRAO [12-15]. No case of BRAO due to cat scratch disease has been recorded in our series of examinations.

Behçet’s disease is a leading cause of uveitis along the Old Silk Road, including North African countries [23]. Behçet’s uveitis typically presents in the form of panuveitis associated with retinal periphlebitis that may be complicated with BRVO [23]. However, retinal artery involvement causing BRAO can also occur in the setting of Behçet’s uveitis [16].

Inflammatory BRAO usually results in visual field defect without or with impairment of VA. The diagnosis of BRAO primarily relies on clinical examination, but related clinical features might be overlooked or misdiagnosed as signs of retinochoroidal inflammation. FA is very useful in establishing a definitive diagnosis of inflammatory BRAO, particularly in subclinical cases or those with a challenging diagnosis.

Several mechanisms might be involved in the development of BRAO associated with posterior uveitis. BRAO may occur at the site of an active focus of retinitis or retinochoroiditis. It could result from a direct compression of the artery by the focus of retinitis or chorioretinitis, leading to interruption of the blood flow. Arterial occlusion near active inflammatory foci may also be explained by arteriolar contraction associated with increased blood viscosity and inhibition of coagulation due to heparin release from the mast cells as a response to an acute inflammatory stimulus [5].

In the absence of the focus of retinitis or chorioretinitis, BRAO could be due to perivasculitis resulting from the infiltration of the vessel wall by the inflammatory cells, which may cause its thickening and as the consequence, disruption of blood flow and arterial thrombosis [14].

In patients with IRVAN syndrome, BRAO have been reported following laser photocoagulation of the aneurysms [19,20]. To the best of our knowledge, only one case of IRVAN syndrome complicated with a primary BRAO similar to our case has been previously reported [21]. The location of macroaneurysm at the junction of the retinal artery tree might make it prone to spontaneous thrombosis.

The cilioretinal artery occlusion in our patient with Behçet’s uveitis probably was functional in nature. An increase of the intraluminal pressure in the retinal capillaries due to the BRVO which is exceeding the pressure in the cilioretinal artery could lead to its occlusion [16].

Resolution of the ocular inflammation and reperfusion of the occluded artery occurred in all patients following anti-infectious and/or systemic corticosteroid therapy. Improvement of VA was recorded in all patients; however, the retinal function did not fully recover in cases with BRAO involving the macula with persistent field defects probably due to the atrophy of the retinal neuroepithelium or macular ischemia. Early and appropriate treatment with anti-inflammatory and/or anti-infectious medication may help induce prompt resolution of the ocular inflammation and reperfusion of the occluded artery and improve visual outcome.

## Conclusions

In conclusion, BRAO may occur in association with an array of infectious or non-infectious posterior uveitis entities, dominated by toxoplasmic retinochoroiditis. It usually causes visual field defect without or with VA impairment. A careful clinical examination, complemented with FA, is mandatory in order not to miss a diagnosis of inflammatory BRAO. The role of medical treatment on the course of the occlusive event requires further investigation.

## Methods

This is a retrospective study including 18 eyes of 18 patients with BRAO associated with posterior uveitis. All patients were examined at the Department of Ophthalmology, Fattouma Bourguiba University Hospital, Monastir, Tunisia between 1998 and 2011. The study was approved by the ethics committee of our institution.

The patients underwent a complete ophthalmic evaluation that included measurement of best-corrected VA, slit-lamp examination, and dilated fundus examination. Fundus photography, FA, and visual field testing were performed in all cases. OCT was performed in eight patients. Mean follow-up was 10 months (range, 6 to 24 months).

## Competing interest

The authors declare that they have no competing interests.

## Authors’ contributions

RK, SM, IK, BJ, SBY, and MK, participated in the sequence alignment and in the design of the study, performed the statistical analysis, and drafted the manuscript. RK and MK conceived of the study and participated in its design and coordination. All authors read and approved the final manuscript.
